# Identification and Clinical Application of Immunological Receptors Targeting Mutated Antigens Expressed by Solid Tumors

**DOI:** 10.3390/cancers12102818

**Published:** 2020-09-30

**Authors:** Anna Pasetto

**Affiliations:** Department of Laboratory Medicine, Division of Clinical Microbiology, ANA FUTURA, Karolinska Institutet, 14152 Huddinge, Sweden; anna.pasetto@ki.se

**Keywords:** TCR, CAR, solid tumors, mutated antigens, clinical trials

In the recent years, immunotherapy has achieved impressive results utilizing different approaches, from (chimeric antigen receptor) CAR-T cells directed against CD19 for the treatment of leukemia and lymphomas to the Nobel prize-winning strategy of check-point inhibition for the treatment of several solid tumors. However, the war against cancer is still far from over, and more effective treatments are urgently needed. Some key factors for a successful treatment are the choice of the antigen targeted, the strategic design of the immunological receptors and the feasibility of the overall therapeutic approach in a clinical setting.

## 1. Antigenic Targets in Solid Tumors

Traditionally, an antigen is defined as a molecule capable of generating an immune response. For cancer treatment, the desired immunological response is one that results in complete and specific killing of tumor cells, leaving normal cells virtually unharmed. Among the antigens that have been reported to be immunogenic are tissue differentiation antigens and cancer germline antigens [1], viral antigens [2,3] and neoantigens [4,5,6,7]. Target antigens can also be divided in (Major histocompatibility complex) MHC-dependent and MHC-independent. MHCs are molecular complexes that present peptides derived from intracellular or extracellular origin. The immunological receptor that can recognize antigens presented by the MHC is the T cell receptor (TCR). TCRs are expressed by all T cells and can detect antigens derived from proteins present in all cell compartments, from surface to cytosol and nuclear locations. MHC-independent antigens are all the antigens that do not need to be expressed together with the MHC to be recognized by an immune receptor: examples of these are mostly cell surface proteins, glycoproteins, glycolipids and carbohydrates that are suitable targets for antibodies or chimeric antigen receptor (CAR) generated from single-chain variable fragment (scFvs) [8,9]. The identification of the best target antigen for cancer therapy is still an open debate. For some approaches, such as check-point inhibitors, it is not even necessary to identify potential targets prior to therapy, although the treatment success may also depend on the presence of certain antigens derived from mutated proteins [10].

## 2. Immunological Receptors in Immunotherapy

Among the most successful immunotherapies for solid tumors is adoptive cell therapy of tumor infiltrating lymphocytes (TIL) reinfused into patients after ex-vivo expansion. This approach has been tested on several tumor types such as breast cancer [11] and gastrointestinal cancer [5,12], but the highest clinical response has been in cutaneous melanoma [13]. TIL’s tumor reactivity can be blocked by anti-MHC antibodies, leading to the conclusion that the reactivity is driven by tumor-specific TCRs. The identification of tumor-specific TCRs is becoming a research field on its own, with different strategies being developed to guide the isolation based on the relative frequency of a clonotype in the TIL population [14], or by tetramer binding ability [15,16] or expression of surface markers such as PD-1 [17,18]. 

For MHC-independent antigens, a traditional approach is to generate a CAR, a molecule that combines different functionalities in different domains. The antigen recognition domain is usually derived from the variable regions of a monoclonal antibody that is linked to an intracellular signaling domain capable of inducing T cell activation: usually, the 4-1BB and CD28 signaling domains serve this purpose [19].

Other interesting chimeric immunological receptors have been designed harnessing ligand–receptor pairs such as cytokines [20] or growth factors such as CD70 [21].

This type of receptor is designed to be expressed by T cells and, therefore, administered in the contest of adoptive therapy of genetically modified cells; to date, two CAR-T cell therapies targeting CD19 are approved for clinical use in the treatment of leukemia and lymphomas [22,23] with impressive results.

Adoptive cell transfer of genetically modified cells is not the only strategy that has been proposed for the treatment of solid cancers. One interesting example is immune-mobilizing monoclonal TCRs against cancer (ImmTACs) where a soluble TCR is coupled with an scFv anti-CD3 domain, and these receptors can engage an MHC-dependent antigen on cancer cells and stimulate cytolytic activity from surrounding TIL by CD3 crosslink [24]. 

## 3. Challenges for Clinical Application

Cancer is a major public health problem and, despite the promising results of approved immunotherapies such as checkpoint inhibitors and CAR-T cells, most patients do not benefit or even have access to these treatments. Several challenges have been identified concerning the design of an efficacious therapy—for example, how to gain access to the solid tumor mass and revert to the immune suppressive tumor microenvironment—but equally important challenges are also found in the process of manufacturing and distributing these new immunotherapies. Highly sophisticated approaches often require very expensive methods that are also difficult to distribute across multiple production centers. The future of immunotherapy relies on new strategic solutions that will be able to combine effective clinical results with treatment feasibility on a larger scale.
